# Nucleated red blood cells explain most of the association between DNA methylation and gestational age

**DOI:** 10.1038/s42003-023-04584-w

**Published:** 2023-02-27

**Authors:** Kristine L. Haftorn, William R. P. Denault, Yunsung Lee, Christian M. Page, Julia Romanowska, Robert Lyle, Øyvind E. Næss, Dana Kristjansson, Per M. Magnus, Siri E. Håberg, Jon Bohlin, Astanand Jugessur

**Affiliations:** 1 Centre for Fertility and Health, Norwegian Institute of Public Health, Oslo, Norway; 2 Institute of Health and Society, University of Oslo, Oslo, Norway; 3 Department of Human Genetics, University of Chicago, Chicago, IL 60637 USA; 4 Department of Physical Health and Ageing, Division of Mental and Physical Health, Norwegian Institute of Public Health, Oslo, Norway; 5 Department of Global Public Health and Primary Care, , University of Bergen, Bergen, Norway; 6 Department of Medical Genetics, Oslo University Hospital and University of Oslo, Oslo, Norway; 7 Division of Mental and Physical Health, Norwegian Institute of Public Health, Oslo, Norway; 8 Department of Genetics and Bioinformatics, Norwegian Institute of Public Health, Oslo, Norway; 9 Division for Infection Control and Environmental Health, Department of Infectious Disease Epidemiology and Modelling, Norwegian Institute of Public Health, Oslo, Norway

**Keywords:** DNA methylation, Erythropoiesis

## Abstract

Determining if specific cell type(s) are responsible for an association between DNA methylation (DNAm) and a given phenotype is important for understanding the biological mechanisms underlying the association. Our EWAS of gestational age (GA) in 953 newborns from the Norwegian MoBa study identified 13,660 CpGs significantly associated with GA (p_Bonferroni_<0.05) after adjustment for cell type composition. When the CellDMC algorithm was applied to explore cell-type specific effects, 2,330 CpGs were significantly associated with GA, mostly in nucleated red blood cells [nRBCs; n = 2,030 (87%)]. Similar patterns were found in another dataset based on a different array and when applying an alternative algorithm to CellDMC called Tensor Composition Analysis (TCA). Our findings point to nRBCs as the main cell type driving the DNAm–GA association, implicating an epigenetic signature of erythropoiesis as a likely mechanism. They also explain the poor correlation observed between epigenetic age clocks for newborns and those for adults.

## Abstract (web summary)

CpG sites associated with gestational age are predominantly found in nucleated red blood cells, which point to an epigenetic signature of erythropoiesis as being partly responsible for this association.

## Introduction

Gestational age (GA) is intimately linked to fetal development. Even slight variations in GA at birth are associated with a wide variety of perinatal health outcomes, some of which have important clinical consequences^1–5^. Epigenetic modifications, such as DNA methylation (DNAm), play a critical role in fetal development^6–8^. DNAm has also been shown to be robustly associated with GA at thousands of CpG sites^5,9–12^. The strong association between DNAm and GA probably reflects biological processes related to fetal development, but the specific mechanisms underlying this association are still unknown. Thus, elucidating these mechanisms may provide a deeper understanding of the molecular processes involved in normal as well as aberrant fetal growth and development.

Most of the previous epigenome-wide association studies (EWASs) of GA were based on DNAm data generated on the Illumina Infinium HumanMethylation450 array (450k) or its predecessor, the Illumina Infinium HumanMethylation27 array (27k)^5,9,12^. These arrays were designed to cover mainly gene promoters and protein-coding regions^13,14^. In December 2015, 450k was replaced by the more comprehensive Illumina Infinium MethylationEPIC array (EPIC), which employs the same technology as 450k for measuring DNAm but contains almost twice the number of CpG sites (~850,000) and has a higher coverage of CpGs in regulatory regions^13^. Despite the substantial improvement in genome-wide coverage of regulatory regions and the higher reproducibility and reliability of EPIC^13^, studies investigating the association between GA and DNAm data generated on EPIC are lacking. It is also uncertain whether the extra regulatory CpGs on EPIC are useful in explaining the association between GA and DNAm.

Most studies exploring the link between DNAm and GA are based on samples from cord blood, which comprises a mixture of cell types^15^. As cell-type proportions vary substantially across individuals and DNAm is highly cell-type specific^16^, it is customary to adjust for cell-type proportions in statistical models in order to avoid bias^17^. Several cellular deconvolution algorithms and cord-blood reference panels are available to infer cell-type proportions from heterogeneous samples and adjust for cord blood cell-type composition in newborn DNAm data^18–20^. However, including cell-type proportions as covariates in the statistical model will not necessarily provide insight as to how cell types influence the association between the explanatory variable and DNAm. One solution is to perform an EWAS in isolated cell types. However, cell sorting of whole-blood samples is costly, especially in large cohort studies with hundreds of thousands of participants.

To counter this, statistical algorithms have been developed to allow the detection of cell-type specific differential DNAm within a heterogeneous mixture of cells without the need for cell sorting or single-cell methods^21–24^. One example is CellDMC, by Zheng et al.^24^, which incorporates interaction terms between the phenotype of interest and the estimated cell-type fractions in a linear modeling framework. Another example is Tensor Composition Analysis (TCA), by Rahmani et al.^23^. which employs matrix factorization to infer cell-type specific DNAm signals that are subsequently used to search for associations in each cell type separately. Exploring cell-type specific associations can be essential to decipher the biological underpinnings of an association between DNAm and a specific phenotype of interest^25^. Whilst changes in cord blood cell-type proportions have been reported for GA^26,27^, studies on cell-type specific epigenetic associations with GA are lacking.

To bridge these knowledge gaps, we investigate the association between cord blood DNAm and GA using an EPIC-derived DNAm dataset comprising 953 newborns and a 450k-derived dataset comprising 1062 newborns. Both datasets are from the Norwegian Mother, Father, and Child Cohort Study (MoBa)^28^. We apply CellDMC to these datasets to determine the relationship between cell-type specific DNAm and GA. We also apply TCA as an alternative method for cell-type-specific analysis. The results show many CpGs associated with GA, predominantly in nucleated red blood cells (nRBCs). This association reflects an epigenetic signature of erythropoiesis in fetal development and provides a biologically plausible rationale for the consistently observed strong association between DNAm and GA. It also helps explain the observed incompatibility between epigenetic age clocks for newborns and those for adults.

## Results

### Study sample characteristics

We analysed cord blood DNAm in newborns from two substudies in MoBa. The main study sample consisted of 953 naturally conceived newborns from the Study of Assisted Reproductive Technology (START), in which DNAm was measured using the EPIC array^29,30^. We also used another dataset consisting of 1062 newborns (referred to as MoBa1 hereafter) with DNAm measured using the 450k array^10^. GA ranged from 216–305 days (mean 280.1 days, SD ± 10.7 days) in START and 209–301 days (mean 279.8 days, SD ± 10.8 days) in MoBa1. Table 1 summarizes the key demographic and clinical characteristics of these two datasets. More MoBa1 mothers continued to smoke during pregnancy compared to START mothers (*p* = 0.033, Table 1). There were also more boys in MoBa1 than in START (*p* = 0.007, Table 1).

**Table 1:** Characteristics of the mothers and newborns in START and MoBa1.

Characteristics	START *n* = 956	MoBa1 *n* = 1062	*p* value^a^
Mothers			
Age (years), mean (SD)	29.9 (4.7)	29.9 (4.3)	0.800
Smoking, *n* (%)			0.033
No smoking before or during pregnancy	478 (50%)	522 (49%)	
Smoked, but quit before pregnancy	245 (26%)	233 (22%)	
Smoked, but quit early in pregnancy	131 (14%)	154 (15%)	
Continued smoking during pregnancy	102 (11%)	153 (14%)	
Newborns			
GA in days, mean (SD)	280.1 (10.7)	279.8 (10.8)	0.400
GA in days, min	216	209	
GA in days, max	305	301	
Birth weight in grams, mean (SD)	3657 (521)	3643 (539)	0.500
Sex (male), *n* (%)	455 (47%)	569 (54%)	0.007

*SD* standard deviation, *GA* gestational age.

^a^Wilcoxon rank-sum test; Pearson’s Chi-squared test.

### Analyses of cell-type composition

**Fig. 1:**
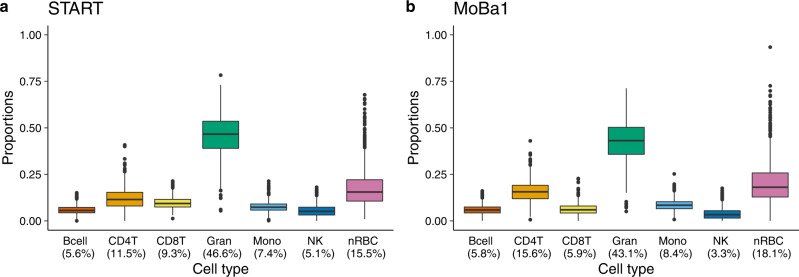
Estimated proportions of seven main cell types in cord blood. **a** Estimated proportions of cell types in the START dataset (*n* = 953, EPIC-based). **b** Estimated proportions of cell types in the MoBa1 dataset (*n* = 1062, 450k-based). The upper and lower box limits correspond to the interquartile range (25 to 75% of the values for each cell type) and the horizontal line in the box represents the median value. The whiskers outstretch 1.5 times the box height from the top and bottom of the box. The dots outside the whiskers represent outliers beyond the interquartile range. The percentage below each cell type denotes the median proportion of that cell type. Bcell B-cell, CD4T CD4 + T-cell, CD8T CD8 + T-cell, Gran granulocyte, Mono monocyte, NK natural killer cell, nRBC nucleated red blood cell.

We estimated the proportion of each of the seven main cell types in cord blood (B-cells, CD4 + T-cells, CD8 + T-cells, granulocytes, monocytes, natural killer cells, and nRBCs) separately in START and MoBa1, using a combined reference dataset consisting of cell-type specific DNAm profiles in cord blood^19^ (Fig. 1 and Supplementary Data 1). As expected from the reference data, granulocytes and nRBCs were the two most abundant cell types in both datasets. The results of a principal component analysis (PCA) of cell-type proportions in START further confirmed that granulocytes and nRBCs explained most of the variance in cell-type composition (Supplementary Fig. 1 and Supplementary Table 1).

We examined the proportion of each cell type in START and found significant correlations with GA in B-cells (Pearson correlation *r* = –0.21, *p* = 6.30 × 10^−11^), CD4 + T-cells (*r* = −0.10, *p* = 0.002), granulocytes (*r* = 0.20, *p* = 5.77 × 10^−10^), and nRBCs (*r* = −0.08, *p* = 0.010; see Supplementary Fig. 2 for more details).

### Conventional EWAS of GA

**Fig. 2:**
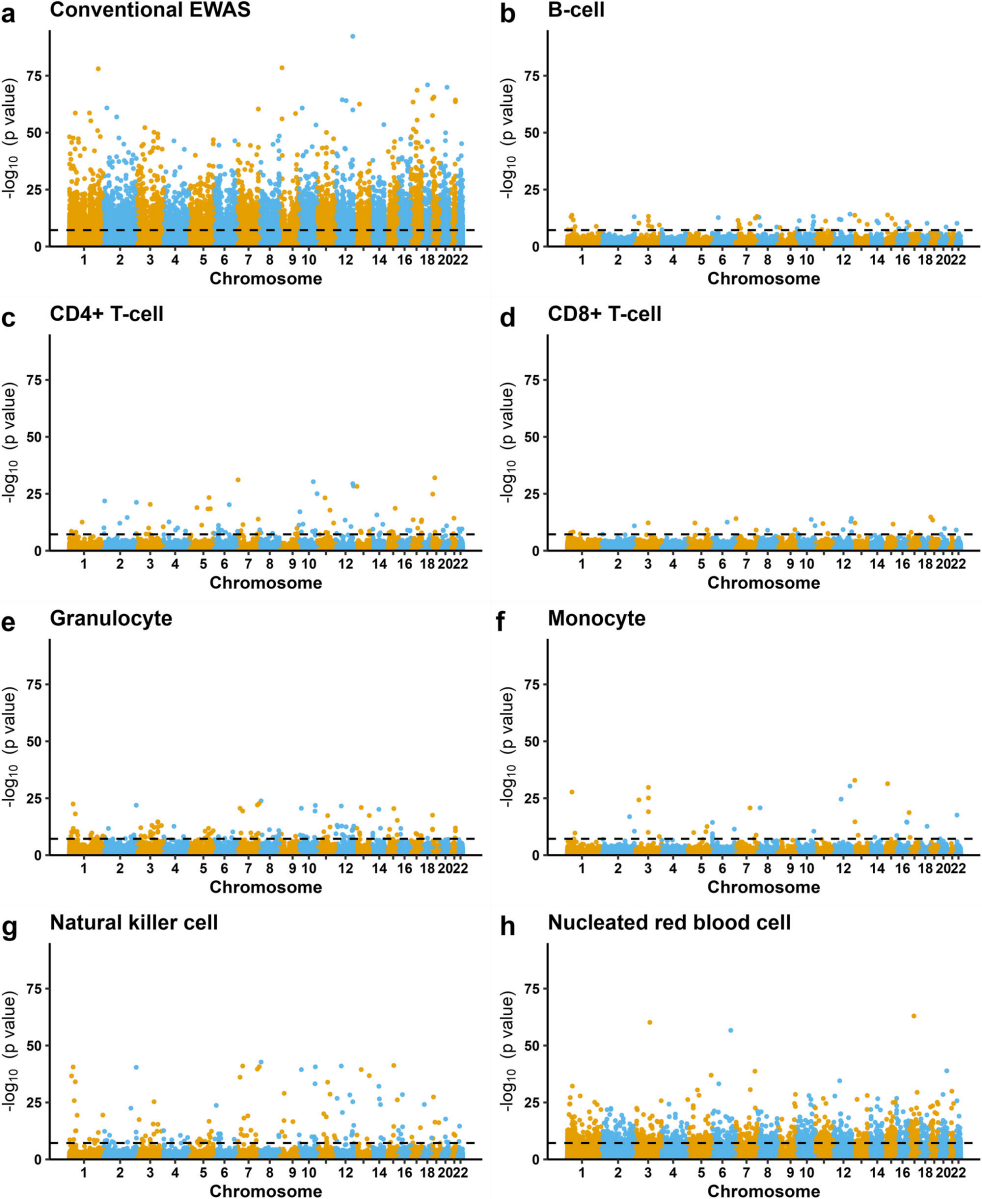
Manhattan plots of the epigenome-wide DNAm associated with GA in START (*n* = 953). **a** Results from the conventional EWAS where we adjusted for the estimated cell-type proportions (see Methods for details of the statistical model). **b**–**h** Results for each of the seven cell types from the cell-type specific analysis using CellDMC. CpG loci are aligned on the x-axis according to their genomic coordinates. The y-axis represents the −log_10_ *p* values. The dashed black line denotes the Bonferroni-corrected genome-wide significance threshold (*p*_B_ < 0.05).

**Fig. 3:**
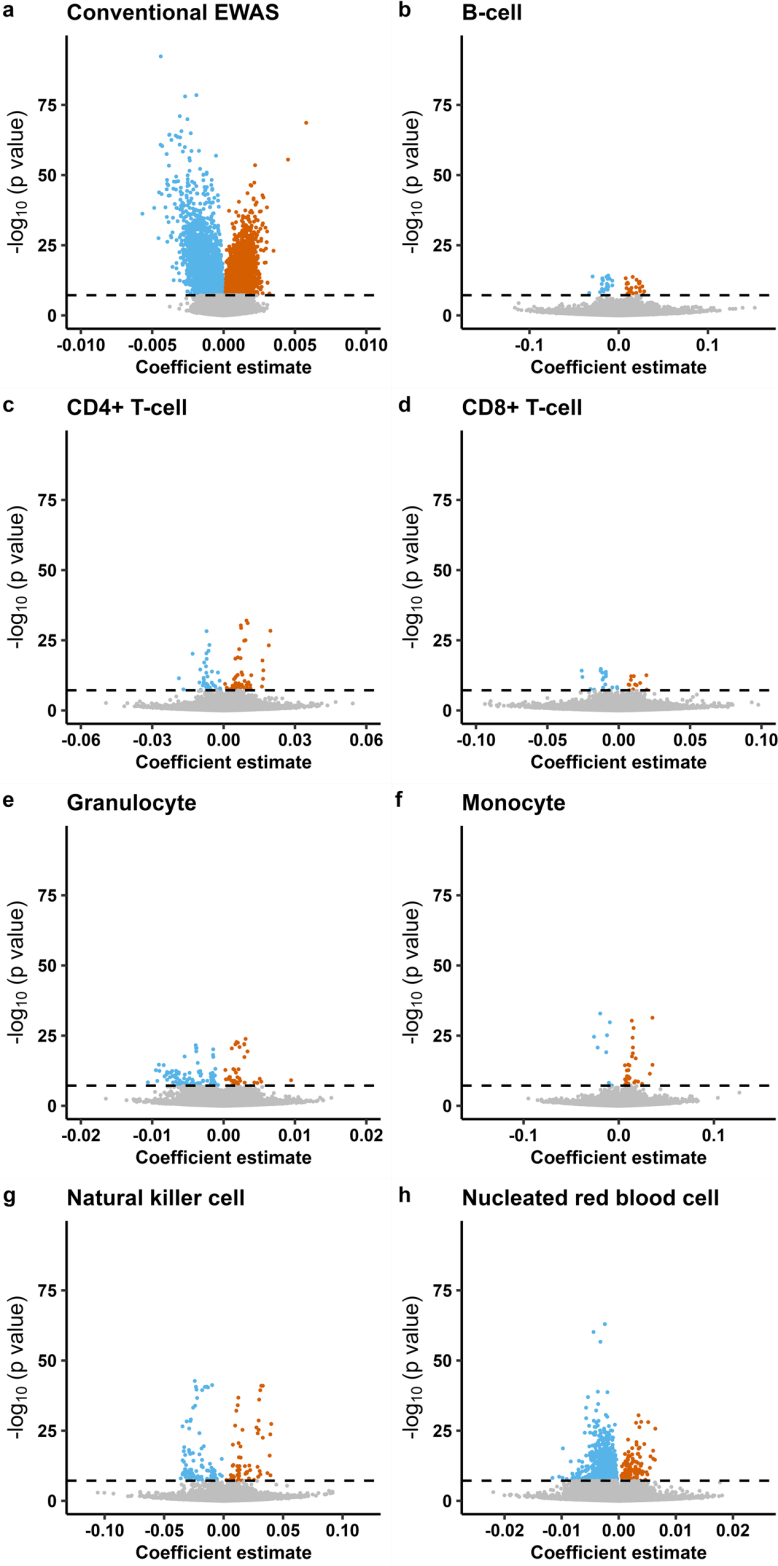
Volcano plots of the epigenome-wide DNAm associated with GA in START (*n* = 953). **a** Results from the conventional EWAS in which we adjusted for estimated cell-type proportions (see Methods for details of the statistical model). **b**–**h** Results for each of the seven cell types from the cell-type specific analysis using CellDMC. Gray dots indicate nonsignificant associations and colored dots indicate those that are Bonferroni-significant (*p*B < 0.05). Blue-colored dots show CpGs with a negative effect size and orange dots show CpGs with a positive effect size. The x-axis represents coefficient estimates (β-values) for the DNAm–GA association, and the y-axis the corresponding -log_10_ *p* values. The horizontal dashed line denotes the Bonferroni-corrected genome-wide significance threshold (*p*_B_ < 0.05).

First, we applied a linear mixed effects regression model to the EPIC-derived START dataset where the outcome was DNAm level at each CpG, the exposure was GA, and the following were included as covariates: cell-type proportions, newborn sex, maternal age, maternal smoking, and batch (see Methods for details). This model is referred to as the conventional EWAS model throughout this paper, since this framework is routinely adopted in the majority of published EWASs. We identified 13,660 CpGs significantly associated with GA after applying a Bonferroni correction for multiple testing (Bonferroni-corrected *p* value (*p*_B_) <0.05, Fig. 2a and Supplementary Data 2). About 7639 (56%) of the GA-associated CpGs were only present on the EPIC array and were distributed across the genome (Supplementary Fig. 3). Most of the GA-associated CpGs in the conventional EWAS were hypermethylated [*n* = 9503 (70%), Fig. 3a].

### Cell-type specific analyses of the association between DNAm and GA

We applied CellDMC to investigate cell-type specific DNAm in the START dataset and identified 2,330 CpGs significantly associated with GA (*p*_B_ <0.05, Fig. 2b–h). Most of these CpGs (*n* = 2030, 87%) were specific for nRBCs (Fig. 2h), and only a few of the CpGs (*n* = 31–157 and 1.3–6.7%) were identified in the other cell types. Moreover, 522 of the 2330 cell-type-specific CpGs associated with GA were also identified in the conventional EWAS. Detailed results of the CellDMC analyses are provided in Supplementary Data 3.

CpGs that were associated with GA in CD4 + T-cells and monocytes were predominantly hypermethylated [CD4 + T-cells: *n* = 67 (65%), Fig. 3c; monocytes: *n* = 29 (78%), Fig. 3f]. We found an almost equal number of hyper- and hypomethylated CpGs associated with GA in B-cells [hypermethylated *n* = 29 (55%); hypomethylated *n* = 24 (45%); Fig. 3b] and CD8 + T-cells [hypermethylated *n* = 13 (42%); hypomethylated *n* = 18 (58%); Fig. 3d]. In contrast, GA-associated CpGs specific for granulocytes, natural killer cells, and nRBCs were predominantly hypomethylated [granulocytes: *n* = 97 (71%), Fig. 3e; natural killer cells: *n* = 97 (62%), Fig. 3g; nRBCs: *n* = 1888 (93%), Fig. 3h].

### Impact of the type of DNAm array: 450k versus EPIC

To determine whether the type of DNAm array had an impact on the cell-type specific results, given the lower coverage of regulatory CpGs on 450k compared to EPIC, we repeated the CellDMC analysis on MoBa1 (*n* = 1062 newborns) in which DNAm was measured using 450k. The results showed a similar pattern of cell-type specific DNAm associated with GA, despite fewer significant CpGs overall (*n* = 373, *p*_B_ < 0.05, Supplementary Data 4 and Supplementary Fig. 4). Specifically, 62% (*n* = 231) of the Bonferroni-significant CpGs mapped to nRBCs.

**Fig. 4:**
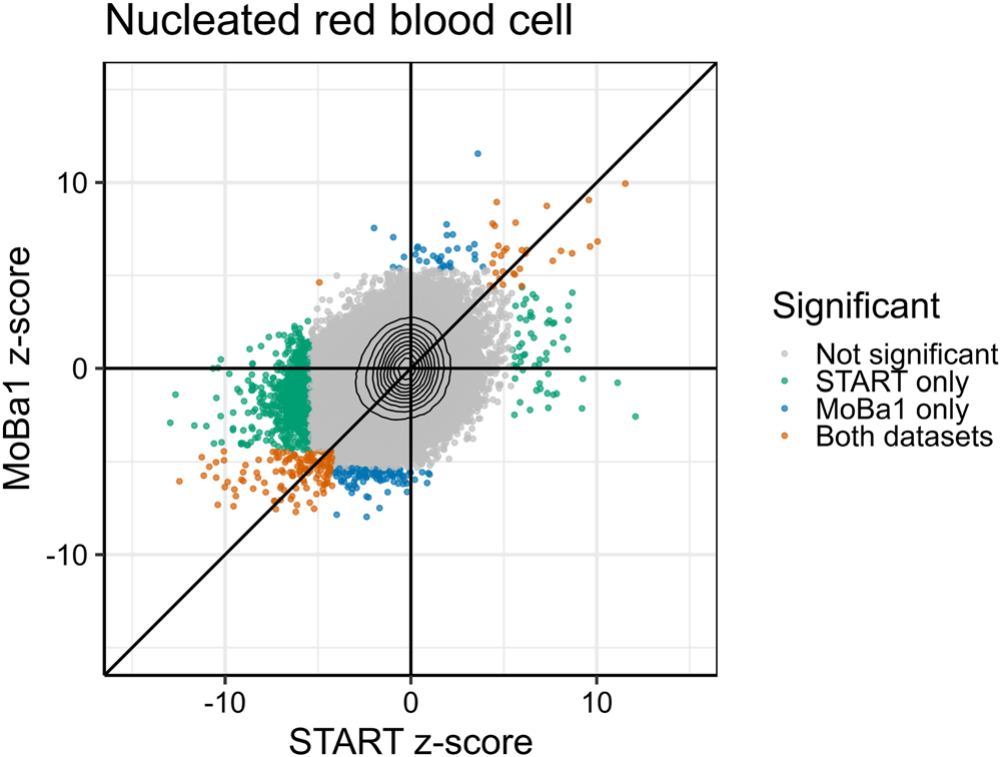
Comparison of nRBC-specific CpGs associated with GA in the EPIC-based START dataset (*n* = 953) and the 450k-based MoBa1 dataset (*n* = 1062). Gray dots indicate nonsignificant CpGs, blue dots CpGs significantly associated only in MoBa1 (*p*_B_ < 0.05), green dots CpGs significantly associated only in START (*p*_B_ < 0.05), and orange dots CpGs significantly associated in both datasets (*r* < 0.05). Black isolines indicate the density of the points, increasing towards the crossing point of the axes. The x and y axes represent *z*-scores (i.e., the coefficient estimate divided by the standard error) for START and MoBa1, respectively.

To further assess the robustness of our findings, we used the *r* value approach of ref. ^31^ to compare the results from START and MoBa1. This approach tests if a CpG is significantly associated in two separate studies and then computes the corresponding false discovery rate (FDR) value of this test, which is referred to as the *r* value (see Methods for details). If the *r* value was <0.05, we deemed a GA–CpG association detected in START as successfully replicated in MoBa1. Among 1129 nRBC-specific CpGs detected in START that were also available on the 450k array, 174 CpGs were significantly replicated in MoBa1 (*r* < 0.05, Fig. 4 and Supplementary Data 5). The results were also consistent in terms of the direction of effect, except for one CpG (cg13746414). Importantly, there was no overlap in CpGs between START and MoBa1 for the remaining six cell types (*r* < 0.05, Supplementary Fig. 5).

### Validation with a different cell-type specific method

To further validate the cell-type specific associations between DNAm and GA, we applied TCA to the START dataset using two different approaches. First, we applied a one-stage implementation of TCA which runs marginal conditional tests for each cell type, analogous to CellDMC. We then applied a two-stage implementation of TCA, by first extracting the cell-type tensors additionally adjusted for the above-mentioned covariates and then performing separate EWAS regressions on each tensor with respect to GA. With the one-stage approach, we identified 979 GA-associated CpGs (*p*_B_ <0.05), whereas with the two-stage approach, we identified 4714 GA-associated CpGs (*p*_B_ <0.05). Both approaches map most of the cell-type specific significant CpGs to nRBCs [*n* = 836 (85%) in the one-stage approach (Supplementary Fig. 6) and *n* = 3130 (66%) in the two-stage approach (Supplementary Fig. 7)]. For all cell types, more CpGs were statistically significant using the two-stage approach compared to the one-stage approach. In granulocytes specifically, 1668 CpGs were identified as significantly associated with GA, of which 829 were also mapped to nRBCs. The results from the one-stage and two-stage TCA analyses can be found in Supplementary Data 6 and 7, respectively.

Among the 2030 nRBC-specific CpGs detected by CellDMC, 623 CpGs were also detected when applying the one-stage TCA (Supplementary Fig. 8). Overall, 260 nRBC-specific CpGs were detected by both CellDMC and the two-stage TCA approach (Supplementary Fig. 9). The results from the one-stage TCA analysis were also generally consistent with those of the CellDMC analysis for the other six cell types (Supplementary Fig. 8), while the two-stage TCA results showed more divergent associations for the other cell types (Supplementary Fig. 9).

### Location of GA-associated CpGs

**Fig. 5:**
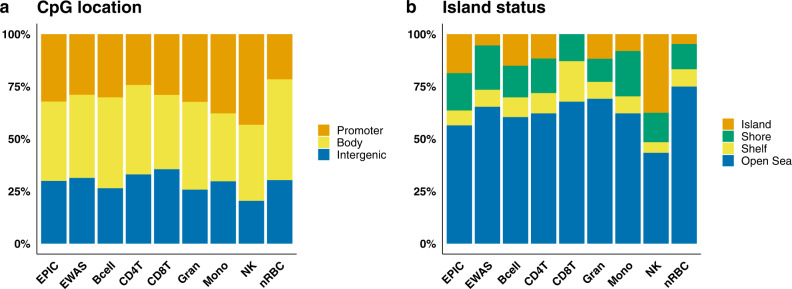
Position enrichment results of CpGs associated with GA compared to all CpGs on the EPIC array. Position enrichment results of all the CpGs on the EPIC array (*n* = 770,586; denoted as EPIC on the x-axis), those specifically associated with GA in the conventional EWAS (*n* = 13,660; EWAS), and each cell type in the CellDMC analyses in START (Bcell, *n* = 53; CD4T, *n* = 103; CD8T, *n* = 31; Gran, *n* = 136; Mono, *n* = 37; NK, *n* = 157; nRBC, *n* = 2030). **a** The proportion of CpGs in the promoter (orange), gene body (yellow), and intergenic (blue) regions. **b** The proportion of CpGs in CpG islands (orange), shores (green), shelves (yellow), and open sea (blue). Bcell B-cell, CD4T CD4 + T-cell, CD8T CD8 + T-cell, Gran granulocyte, Mono monocyte, NK natural killer cell, nRBC nucleated red blood cell.

We scrutinized the GA-associated CpGs identified by the conventional EWAS and CellDMC analyses according to their location in the genome (Fig. 5 and Supplementary Data 2 and 3). The 2030 nRBC-specific CpGs that were significantly associated with GA in START were predominantly localized to gene bodies (48% of the nRBC-specific CpGs versus 30% of all CpGs on EPIC, *p* = 2.5 × 10^−67^, Fig. 5a), open sea (75% versus 56%, *p* = 2.2 × 10^−69^, Fig. 5b), and CpG island shelves (8.2% versus 7.1%, *p* = 0.023, Fig. 5b). Markedly fewer nRBC-specific CpGs were in promoter regions (22 versus 38%, *p* = 2.8 ×10^−55^, Fig. 5a), shores (12 versus 18%, *p* = 1.0 × 10^−12^, Fig. 5b), and CpG islands (4.7% versus 19%, *p* = 5.3 × 10^−77^, Fig. 5b). We discovered a similar pattern of CpG localization in the nRBC-specific MoBa1 results. The corresponding patterns for the other cell types showed more variation between the two datasets (Supplementary Fig. 10), which may be due to a substantially lower number of CpGs in each category.

### Gene annotation and enrichment analysis of nRBC-specific CpGs associated with GA

We used the online Genomic Regions Enrichment of Annotations Tool (GREAT)^32^ to examine whether the 2030 GA-associated CpGs for nRBC were located near or within any gene of known pathway annotation. 2836 genes were identified using this approach (Supplementary Data 8), 198 of which were associated with more than three differentially methylated CpGs. A foreground/background hypergeometric test was performed on the 2030 GA-associated nRBC-specific CpGs. The results of this test revealed four clusters of Gene Ontology (GO) biological processes significantly enriched in our data (Supplementary Data 9). These processes were related to (i) response to corticosteroid (75 CpGs/55 genes, *p*_B_ = 0.0001), (ii) response to purine-containing compound (65 CpGs/45 genes, *p*_B_ = 0.002), (iii) granulocyte migration (34 CpGs/23 genes, *p*_B_ = 0.006), and (iv) stress-activated protein kinase signaling cascade (58 CpGs/32 genes, *p*_B_ = 0.01). When the analyses were restricted to only those CpGs that are present on both 450k and EPIC, we did not find any significantly enriched biological pathways.

## Discussion

Although epigenome-wide associations between GA and DNAm in cord blood are now well established, little is known about the contribution of different cell types and the biological mechanisms underlying these associations. In this study, we explored the association between GA and DNAm using data from two types of DNAm arrays (EPIC and 450k) and conducted both a conventional EWAS as well an investigation of cell-type specific associations. We found that most of the cell-type-specific associations between DNAm and GA were restricted to nRBCs. These results were robust across different datasets, DNAm arrays, and analysis methods. Our results point to a strong link between red blood cell development (erythropoiesis) in fetal life and fetal growth as measured by GA, providing critical insights and implications for further studies on the relationship between DNAm and GA.

In the conventional EWAS, we identified 13,660 CpGs linked to 8669 genes as being differentially methylated with GA. Slightly more of the significant CpGs were specific for the EPIC array (56%), despite only 48% of the CpGs being EPIC-specific. Bohlin et al.^10^ previously applied a similar model to the MoBa1 dataset and identified 5474 CpGs associated with GA. 2556 of the CpGs and 1741 of the genes identified in that study overlap with our results in the START EPIC-based dataset. We also compared our results to the “all births model” from a recent meta-analysis by Merid et al.^5^ where the authors investigated GA and DNAm measured on 450k in cord-blood DNA from 6885 newborns in 20 different cohorts. The authors identified 17,095 CpGs significantly associated with GA, of which 4688 CpGs and 4437 genes overlap with our results. Of note, MoBa1 and yet another MoBa-based dataset (MoBa2) were also included in the meta-analysis by Merid et al. Nevertheless, these comparisons show that the results from our conventional EWAS model are concordant with those of previous studies on DNAm and GA.

As a primary step to explore cell-type specific changes in DNAm with GA, we used the interaction-based algorithm CellDMC that has been validated in several EWAS datasets and data in which the actual cell-type composition is known^24,33,34^. We identified 2330 differentially methylated CpGs associated with GA, with an overwhelming number of the significant CpGs confined to nRBCs (2030 CpGs linked to 2836 genes). This is particularly striking given that nRBCs are not the dominant cell type in terms of variation and abundance. Taken together, these findings strongly suggest that DNAm changes in nRBCs are responsible for the observed DNAm–GA association.

It is nevertheless important to account for the limited sensitivity of CellDMC when including seven different cell types in the analysis^33^. To assess this limited sensitivity and verify that the nRBC-specific results were not an array-based artifact, we repeated the CellDMC analyses in MoBa1, which is a 450k-based dataset stemming from the same source population as the START dataset (MoBa). We observed a similar pattern of cell-type-specific association with GA as with the START dataset, although there were fewer significant CpGs in the MoBa1 dataset. Moreover, 174 nRBC-specific CpGs were significantly associated with GA in both datasets, as opposed to no such overlap in CpGs across the other six cell types. One option to further increase the power of the CellDMC analysis would have been to merge the two datasets over the common set of 450k CpGs. Even though this would have increased the sample size substantially, such an approach has several major drawbacks. First, one would lose the much greater coverage of the EPIC array and possibly miss important associations between GA and CpGs that are only detectable using EPIC-derived DNAm data. Second, merging the datasets would introduce a new batch variable that would need to be accounted for in the model. We thus opted to keep the analyses of the two datasets separate.

To further validate our results, we applied another method for cell-type specific analysis, TCA, to the START data. TCA utilizes a statistical framework based on matrix factorization^23^. The results from both the one-stage and two-stage applications of TCA showed a similar pattern of cell-type specific association with GA as observed with CellDMC. Our findings are also consistent with a previous study on nRBCs pointing to extensive DNAm changes in nRBCs between preterm and term newborns^35^. In that study, the authors identified 9258 differentially methylated sites when comparing nRBCs from preterm and term newborns. These sites were predominantly hypomethylated and enriched in gene body and intergenic regions^35^. Taken together, these results strengthen the interpretation that nRBCs are the primary cell type driving the association between DNAm and GA in cord blood.

nRBCs are an integral part of erythropoiesis, the process by which mature red blood cells (erythrocytes) are produced in adult and fetal bone marrow, fetal liver, and the embryonic yolk sac. Erythropoiesis is crucial for embryonic and fetal growth. During the third trimester of pregnancy, the production of erythrocytes is approximately three to five times that of the adult steady-state levels^36^. Although nRBCs circulate in the fetal bloodstream throughout pregnancy, they stay in circulation for only a few days after birth^37^. Several genes annotated to the nRBC-specific CpGs that we found to be associated with GA are implicated in a wide array of biological processes involved in erythropoiesis. A subset of the genes related to these processes are described in more detail in Supplementary Data 10. Briefly, these processes include cell-cycle progression and cytokinesis^38,39^, chromatin condensation^39,40^, hemoglobin synthesis^38^, mitochondrial function and iron metabolism^38,41,42^, degradation of proteins and organelles^34,43^, erythroblastic island formation^44^, and enucleation^39,40^. Moreover, several of the genes are essential for the switch from fetal to adult hemoglobin, which occurs shortly after birth^45^. Taken together, our findings provide strong support for fetal erythropoiesis representing an important biological mechanism underlying the association between DNAm and GA.

To learn more about the mechanisms contributing to the nRBC-specific association between DNAm and GA, we searched for the enrichment of specific biological pathways in the set of nRBC-specific CpGs. One of the main clusters of biological pathways was the response to corticosteroids, and more specifically, the response to glucocorticoids. Glucocorticoids are a class of corticosteroids that are essential for a wide variety of biological processes, including proliferation, differentiation, and apoptosis of many cell types in response to stress. They also play a pivotal role in pregnancy and normal fetal development^46^, even though prenatal overexposure to glucocorticoids has also been reported to be detrimental to fetal growth and postnatal physiology^47,48^. Glucocorticoids are known regulators of erythroid progenitors^49,50^, and the glucocorticoid receptor encoded by *NR3C1* controls several processes involved in erythropoiesis^51–53^. In particular, the glucocorticoid receptor controls erythroid response to stress^54–56^. Stress, such as hypoxia, leads to the glucocorticoid receptor-dependent activation of the *BMP4*-dependent stress erythropoiesis pathway, in which many new erythrocytes are generated to maintain homeostasis^57^. Interestingly, stress erythropoiesis shares several similarities with fetal erythropoiesis^58^.

The link between erythropoiesis and GA is not unprecedented. Several of the genes found to be relevant for erythropoiesis in our data have previously been identified in other studies of GA. A few examples include *NCOR2*^5,10,59,60^, *HDAC4*^5,10,60^, *CASP8*^5,10,60,61^, and *RAPGEF2*^5,60,62^. The nuclear receptor co-repressor encoded by *NCOR2* interacts with the transcription factor BCL11A in regulating the expression of fetal hemoglobin^63^. NCOR2 also promotes chromatin condensation, which is a crucial step during terminal erythropoiesis. Histone deacetylase 4 (*HDAC4*) also plays a key role in chromatin condensation and associates directly with the key erythroid transcription factor GATA1^64^. *CASP8* encodes the protease Caspase 8, which is a key activator of effector caspases required for terminal erythroid differentiation^65^. Finally, *RAPGEF2* encodes a guanine nucleotide exchange factor known to play an important role in embryonic hematopoiesis^66^.

The results of our study, as well as those of others described above, strongly suggest that DNAm patterns related to erythropoiesis are at least partly responsible for the observed association between DNAm and GA. Our findings of predominantly hypomethylated nRBC-specific CpGs are in line with previous studies showing progressive global DNA hypomethylation involved in erythroid lineage commitment and differentiation as well as chromatin condensation and enucleation of nRBCs during erythropoiesis^67,68^. Other studies have consistently shown a higher proportion of hypomethylated CpGs amongst those associated with GA^5,10,59,61^.

Further, the findings that nRBCs are the primary drivers behind the association between DNAm and GA may help explain the poor correlation observed between epigenetic clocks for newborn GA and those for chronological age in adults^10,11^. Indeed, GA-related changes in cord blood DNAm do not persist through childhood and adolescence, as shown in a longitudinal analysis of DNAm associated with GA^59^ and a meta-analysis of several EWASs of GA^5^. This could be due to the rapid loss of nRBCs with increasing GA and its subsequent disappearance from the bloodstream of healthy newborns within the first few days after birth. In other words, the disappearance of nRBCs shortly after birth implies that the main driver behind the GA-related changes in cord blood DNAm also disappears. Moreover, the association between GA and specific DNAm changes in nRBCs, as demonstrated by our study, may also help explain why GA acceleration (GAA, defined as the discrepancy between GA predicted from DNAm data and GA determined by clinical measurements) has been linked to several adverse outcomes^11,69,70^. In this regard, it is interesting to note that increased nRBC counts at birth are associated with a higher risk of mortality and adverse neonatal outcomes and have been suggested as a predictive marker for perinatal hypoxia, intrauterine growth restriction, and preeclampsia^71–75^. Further studies are needed to determine if GAA is indeed related to these or other adverse outcomes, and if differences in nRBCs may be driving these associations.

The results of our study may have important clinical implications. For instance, fetal nRBCs are routinely isolated from the mother’s peripheral blood during pregnancy for prenatal diagnostics, and several experimental approaches are available for the rapid isolation of nRBCs^76,77^. Our findings may help pave the way for the development of DNAm-based GA prediction during pregnancy based on nRBC-specific assays, which may provide a more targeted assessment of fetal growth and prenatal development.

One important limitation of our study is the use of in silico estimations of cell-type proportions. Although we have used a reference-based method with validated cord blood-specific reference data, it is important to bear in mind that the proportions we have used here are only estimates. In addition, since the cell-type proportions are essentially fractions that sum up to one, they are not independent of each other, and the correlation between them may impact our analyses. However, since our results were robust despite the use of different DNAm arrays, datasets, and methods, our findings are unlikely to be severely affected by these limitations.

In conclusion, the results of our study strongly indicate that nRBCs are the primary drivers behind the observed DNAm–GA association. Importantly, an epigenetic signature of erythropoiesis seems to be partly responsible for this association, providing a biologically compelling mechanism that links GA, DNAm, and nRBCs. Furthermore, our findings provide an explanation for the poor correlation observed between epigenetic clocks for newborn GA and those for chronological age in adults, contributing important mechanistic insights into the epigenetic regulation of fetal growth and development.

## Methods

### Study population

MoBa is a population-based pregnancy cohort study in which ~114,500 newborns, 95,200 mothers, and 75,200 fathers were recruited from all over Norway from 1999 to 2008^28^. The mothers consented to participation in 41% of the pregnancies. The study participants have been followed at different time points via self-administered questionnaires and linkage to the Medical Birth Registry of Norway (MBRN). Further details on MoBa have been provided in our previous publications^28,78^.

For this study specifically, we used two non-overlapping subsamples: (i) the Study of Assisted Reproductive Technology (START; *n* = 953 newborns) and (ii) MoBa1 (*n* = 1062 newborns). Both datasets are based on cord blood samples from the same source population (MoBa). However, they differ in the methylation array used to generate the DNAm data: START used EPIC whereas MoBa1 used 450k (see below for details). An overview of the sample selection and analysis flow is provided in Supplementary Fig. 11. Detailed characteristics and eligibility criteria for the START and MoBa1 datasets have been provided in our previous work^29,79^.

### Sample processing, DNAm measurement, and quality control

The sample processing, DNAm measurement, and quality control pipeline used for data cleaning have been extensively detailed in our previous works^29,79^. Briefly, cord blood samples taken by a midwife immediately after birth were frozen. For the START dataset, DNAm was measured at 885,000 CpG sites using the Illumina Infinium MethylationEPIC BeadChip (Illumina, San Diego, USA). The raw iDAT files were processed in four batches using the R package *RnBeads*^80^. Cross-hybridizing probes^81^ and probes that had a detection *p* value above 0.01 were removed using the greedycut algorithm in *RnBeads*. We also excluded probes in which the last three bases overlapped with a single-nucleotide polymorphism (SNP). The remaining DNAm signal was processed using BMIQ^82^ to normalize the type I and type II probe chemistries^83^. The RnBeads output of control probes were visually inspected for all samples, and those with low overall signals were removed. The greedycut algorithm was used to remove outliers with markedly different DNAm signals than the rest of the samples, resulting in the removal of 58 samples in total. For consistency, CpG sites excluded from one batch due to poor quality and low detection *p* value were also removed from all subsequent batches.

For the MoBa1 samples, DNAm was measured at 485,577 CpG sites using the Illumina Infinium HumanMethylation450 BeadChip (Illumina, San Diego, USA). Arrays not fulfilling the 5% detection *p* value were removed together with all duplicates. Within-array normalization was carried out using BMIQ from the *wateRmelon* R package^84^.

### Variables

Information on GA, newborn sex and birth weight, maternal age, parity, and whether the birth was induced was extracted from MBRN. GA at birth was estimated by ultrasound measurements around week 18 of pregnancy. Since newborn sex may occasionally be incorrectly recorded in MBRN, we inferred sex from the DNAm data. As a result, one female was reclassified as male, and five males were reclassified as females. Information on maternal smoking was derived from the MoBa questionnaires and was included as a four-level categorical variable: (i) no smoking before or during pregnancy; (ii) smoked, but quit before pregnancy; (iii) smoked, but quit early in pregnancy; and (iv) continued smoking during pregnancy.

### Estimation of cell-type proportions

To estimate cell-type proportions in our samples, we used the filtered and combined reference dataset “FlowSorted.CordBloodCombined.450 k” from ref. ^19^, which specifies seven main cell types in cord blood (B-cells, CD4 + T-cells, CD8 + T-cells, granulocytes, monocytes, natural killer cells, and nRBCs). We used the estimateCellCounts2 function in the *FlowSorted.Blood.EPIC* R package^85^ and the IDentifying Optimal Libraries (IDOL) probe selection to perform cellular deconvolution and noob preprocessing.

### Statistics and reproducibility

After quality control, the sample available for the current analyses in the START dataset consisted of 770,586 autosomal CpGs and 953 newborns conceived naturally and for whom we had information on ultrasound-based GA (Supplementary Fig. 9). For the MoBa1 dataset, the sample available for the current analyses comprised 473,731 autosomal CpGs and 1062 newborns with information on ultrasound-based GA (Supplementary Fig. 9).

Principal component analysis (PCA) of estimated cell-type proportions was conducted using the prcomp R function. The R package *robustbase*^86^ for MM-type robust regression was used to assess the relationship between cell-type composition and GA. Bonferroni correction was applied to the results from the conventional EWAS and cell-type-specific models to control for multiple testing. A Bonferroni *p* value (*p*_B_) <0.05 was declared statistically significant.

### Analyses in START

In the conventional EWAS model, we screened for associations between DNAm in cord blood and GA at birth by applying a linear mixed-effect model to each of the 770,586 CpG sites remaining after quality control. The β-values of the individual CpGs were used as the response (dependent) variables and GA was used as the explanatory (independent) variable, with adjustments made for newborn sex, maternal age, maternal smoking, cell-type proportions, and array plate in the regression model.

To assess interactions between cell-type specific DNAm and GA, we performed epigenome-wide analyses using the CellDMC framework as outlined in ref. ^24^ and the corresponding CellDMC function in the EpiDISH R package. Briefly, CellDMC runs a linear model similar to that used in our conventional EWAS, but it also includes an interaction term to inform the model whether there is a significant interaction between the exposure and the corresponding fraction of each specific cell type. Estimates of the regression coefficients and *p* values are calculated for each cell type using least squares. As with the conventional EWAS, newborn sex, maternal age, maternal smoking, and plate were also included as covariates in the CellDMC model. Bonferroni correction was applied to all the results from the conventional EWAS and CellDMC models to control for multiple testing. As before, a Bonferroni *p* value (*p*_B_) <0.05 was declared statistically significant.

Besides CellDMC, we also applied the TCA framework developed by ref. ^23^ to detect cell-type specific DNAm–GA associations. In contrast to CellDMC, TCA is based on the concept of matrix factorization. Specifically, TCA uses the DNAm measurements from the mixed samples along with information on cell-type proportions (in our case, the ones that are estimated) for each individual and calculates a three-dimensional tensor of DNAm values for each cell type in each individual. The TCA framework further allows a search for statistical associations between cell-type specific signals and an outcome or exposure of interest. We used two different approaches for TCA based on the available functions in the *TCA* package^23^. First, we applied a one-stage approach using the tca function, which fits a model for all cell types jointly and tests the effect of each cell type separately for statistical significance. We included the same covariates in the TCA model as in the CellDMC and conventional EWAS models (newborn sex, maternal age, maternal smoking, and array plate). Additionally, we applied a two-stage approach, where a tensor for each cell type is first inferred and then an EWAS of GA is conducted for each tensor. This was carried out by first using the tca function to fit a model including all covariates mentioned above except GA. The model resulting from the tca function was subsequently added as input for the tensor function, obtaining new DNAm tensors for each cell type. An EWAS of GA was then performed for each cell-type-specific tensor.

### Analyses in MoBa1

To test whether array type had an impact on the findings obtained from the analysis of the EPIC-based START dataset, we re-ran the CellDMC analysis on the 450k-based MoBa1 dataset, testing all the 473,731 CpGs available in this dataset.

To compare the CellDMC results from MoBa1 with those from START, we applied the *r* value approach suggested by ref. ^31^, which allows a rigorous assessment of the replication of findings. In short, we tested each CpG for association with GA in both datasets (MoBa1 and START) and computed an *r* value (the lowest FDR level at which the finding was replicated). We chose the *r* value approach over other approaches, such as those used in a standard meta-analysis or a two-step replication study, for the following reasons. First, a meta-analysis tests whether there is any signal across the two studies; however, it does not test whether the two studies show appropriate significance. Second, assessing replicability in a two-step replication study is not straightforward, as this requires adequate control of the type I error in both studies. This may involve a different number of tests, especially as we use two types of DNAm arrays (EPIC and 450k). Thus, the approach of ref. ^31^ provides a simpler solution for assessing replicability and for controlling the type I error.

### Location of CpGs

Information on CpG location and regulatory regions was extracted from the respective Illumina Manifest Files (Infinium MethylationEPIC v1.0 B4 for START and HumanMethylation450 v1.2 for MoBa1). One-tailed hypergeometric tests were conducted to assess the relative enrichment of CpGs in specific regions of interest.

### Gene annotation and enrichment analysis

CpGs were annotated using the online Genomic Regions Enrichment of Annotations Tool (GREAT^32^) using the human genome build hg19 (GRCh37). GREAT was selected amongst other competing methods because it considers both proximal (5.0 kb upstream and 1.0 kb downstream) and distal (up to 1000 kb) regulatory regions. This is an advantage over other methods that only take proximal regions into account, because taking distal regulatory regions into account enables an assessment of the extra information gained from detecting DNAm on distal regulatory CpGs on the EPIC array. For gene enrichment analysis, GREAT performed a foreground/background hypergeometric test over genomic regions using the total number of CpGs surviving quality control as background (770,586 CpGs for the EPIC analyses and 473,731 CpGs for the 450k analyses). Finally, GREAT extracts information from Gene Ontology (GO) and other ontologies covering human and mouse phenotypes^32^.

### Reporting summary

Further information on research design is available in the Nature Portfolio Reporting Summary linked to this article.

## Supplementary information

Supplementary information

Description of Additional Supplementary Files

Supplementary Data 1

Supplementary Data 2

Supplementary Data 3

Supplementary Data 4

Supplementary Data 5

Supplementary Data 6

Supplementary Data 7

Supplementary Data 8

Supplementary Data 9

Supplementary Data 10

Reporting Summary

## Data Availability

Access to the START and MoBa1 DNAm datasets can be obtained by applying to the Norwegian Institute of Public Health (NIPH). Restrictions apply regarding the availability of these data, which were originally used under specific approvals for the current study and are therefore not publicly available. Access can only be given after approval by the Norwegian Regional Committees for Medical and Health Research Ethics (REK) under the provision that the applications are consistent with the consent provided. An application form can be found on the NIPH website at https://www.fhi.no/en/studies/moba/. Specific questions regarding access to data in this study can also be directed to Dr. Siri E. Håberg (Siri.Haberg@fhi.no). The data generated in this study are provided as Supplementary Data.

## Code availability

All statistical analyses were performed using R version 4.1.2^87^. R scripts are available from the authors upon request.
