# Supplementary material for: Nucleated red blood cells explain most of the association between DNA methylation and gestational age
Source: Commun Biol. 2023 Feb 27;6:224. doi: 10.1038/s42003-023-04584-w (PMC9971030; doi:10.1038/s42003-023-04584-w)
Supplement: Supplementary file 1 — Supplementary information [file 42003_2023_4584_MOESM1_ESM.pdf]

**Supplementary information**

Supplement to Haftorn KL, Denault WRP, Lee Y, et al. Nucleated red blood cells explain most of the association between DNA methylation and gestational age

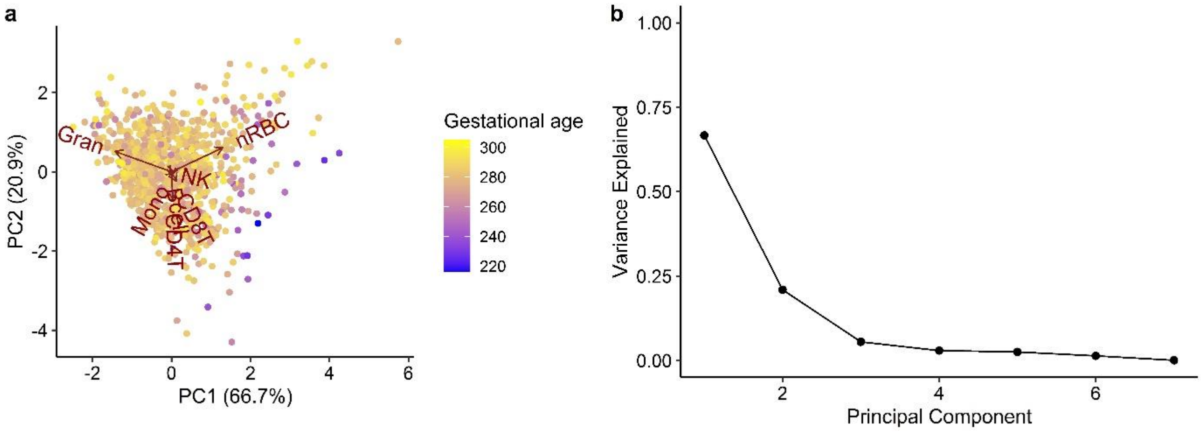


**Supplementary Figure 1. Principal component analysis (PCA) of cell-type composition in the START dataset. a** Shows the first principal component (PC1) against the second (PC2). Each dot represents an individual GA observation (i.e., a singleton newborn), and the colour gradient indicates increasing gestational age (from 220 (blue) to 300 days (yellow)). **b** The percentage of variance explained by the first seven PCs. Abbreviations: Bcell: B-cell; CD4T: CD4+ T-cell; CD8T: CD8+ T-cell; Gran: granulocyte; Mono: monocyte; NK: natural killer cell; nRBC: nucleated red blood cell

**Supplementary Table 1. Loadings of seven cell types for the first seven PCs in a PCA of cell-type composition in the START dataset.**

| **Cell type*** | **PC1** | **PC2** | **PC3** | **PC4** | **PC5** | **PC6** | **PC7** |
|---|---|---|---|---|---|---|---|
| Bcell | 0.011 | -0.098 | 0.279 | 0.020 | 0.133 | -0.895 | 0.307 |
| CD4T | 0.020 | -0.637 | -0.521 | -0.421 | -0.071 | 0.016 | 0.374 |
| CD8T | 0.067 | -0.219 | -0.072 | 0.717 | 0.459 | 0.226 | 0.408 |
| Gran | -0.749 | 0.461 | -0.264 | -0.072 | 0.058 | -0.003 | 0.386 |
| Mono | -0.014 | -0.063 | 0.262 | 0.272 | -0.824 | 0.110 | 0.402 |
| NK | 0.035 | -0.010 | 0.641 | -0.465 | 0.290 | 0.367 | 0.390 |
| nRBC | 0.658 | 0.566 | -0.310 | -0.112 | -0.014 | -0.028 | 0.369 |

*Abbreviations: Bcell: B-cell; CD4T: CD4+ T-cell; CD8T: CD8+ T-cell; Gran: granulocyte; Mono: monocyte; NK: natural killer cell; nRBC: nucleated red blood cell

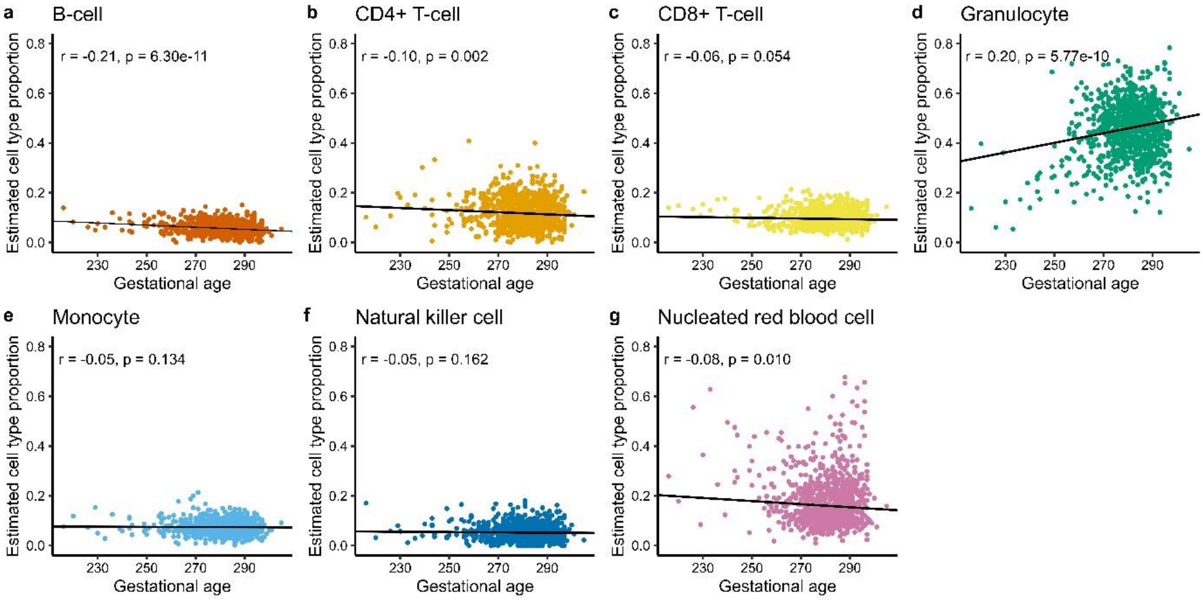


**Supplementary Figure 2. Association of estimated cell-type proportions for seven cell types in cord blood with GA in START**. Pearson correlation (r) estimates and p-values are displayed in each panel. The solid line in each plot is the MM-type robust regression of the estimated cell-type proportion on GA.

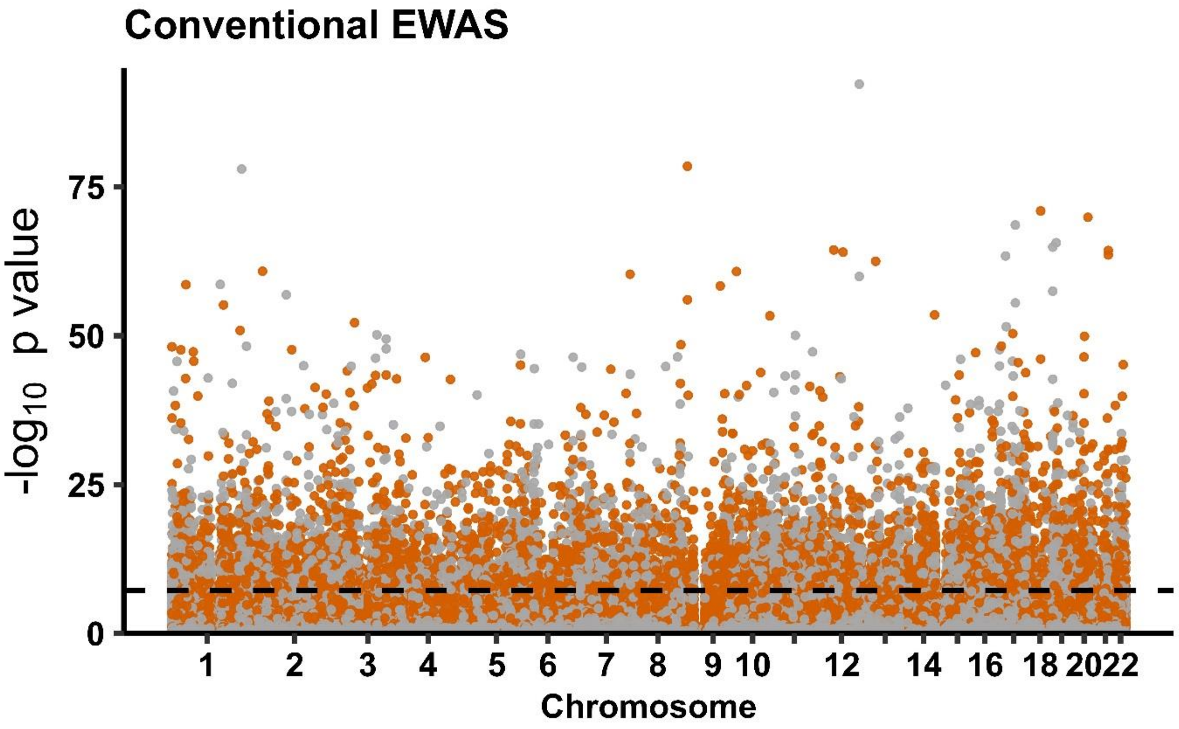


**Supplementary Figure 3. GA-associated CpGs specific for EPIC.** Manhattan plot of the epigenome-wide DNAm associated with GA identified with the 'Conventional EWAS model' in START (see the Methods section in the main text for details). Orange dots represent CpGs only present on EPIC. Grey dots represent CpGs present on both EPIC and 450k. CpG loci are aligned on the x-axis according to their genomic coordinates. The y-axis represents the -log10 p values. The dashed black line denotes the Bonferroni-corrected genome-wide significance threshold ($p_B < 0.05$).

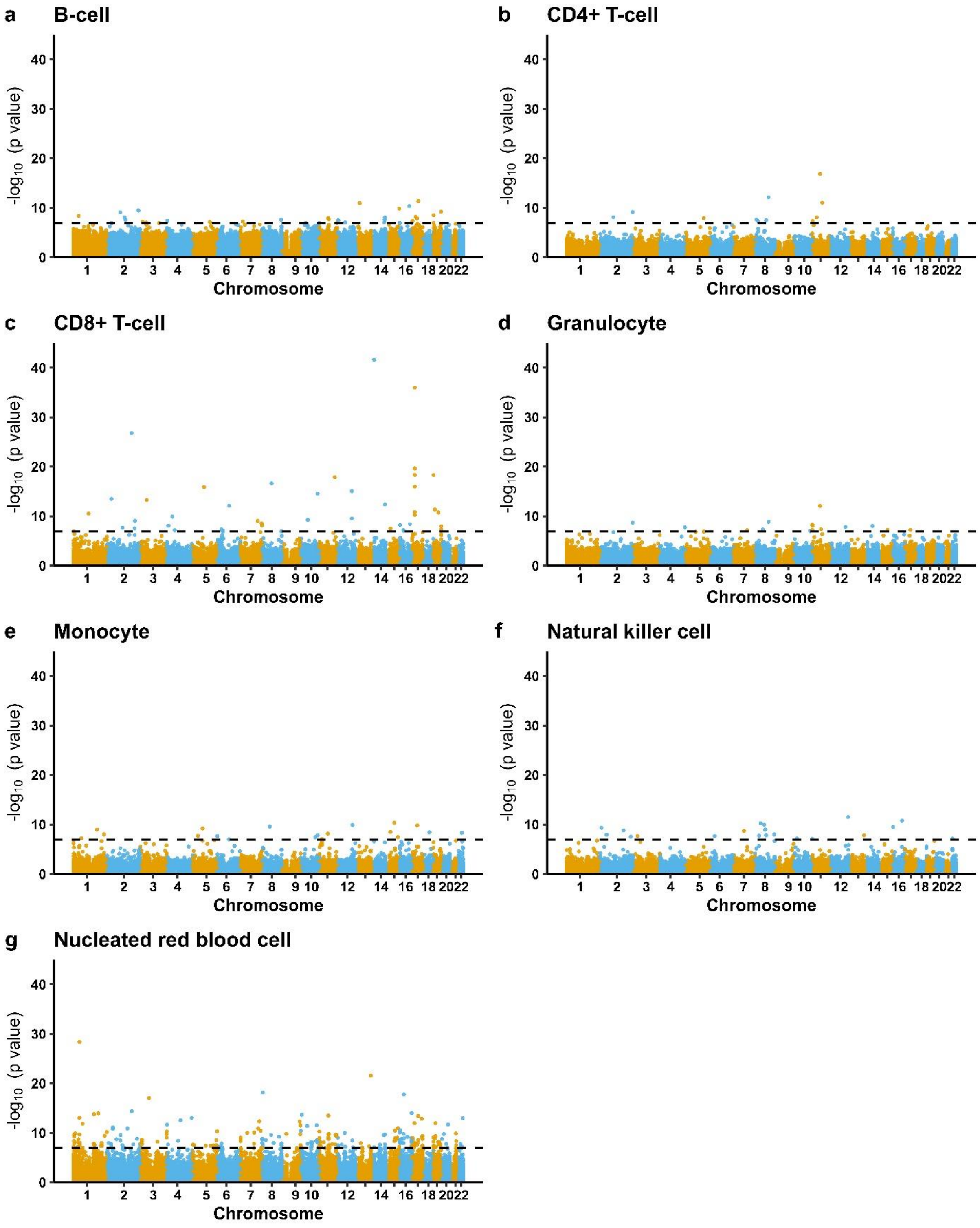


**Supplementary Figure 4. Manhattan plots of the epigenome-wide DNA methylation associated with GA in MoBa1 identified using CellDMC.** Results are shown for each of the seven cell types. CpG loci are aligned on the x-axis according to their genomic coordinates. The y-axis represents the $-\log_{10}$ p values. The dashed black line denotes the Bonferroni-corrected genome-wide significance threshold ($p_B < 0.05$).

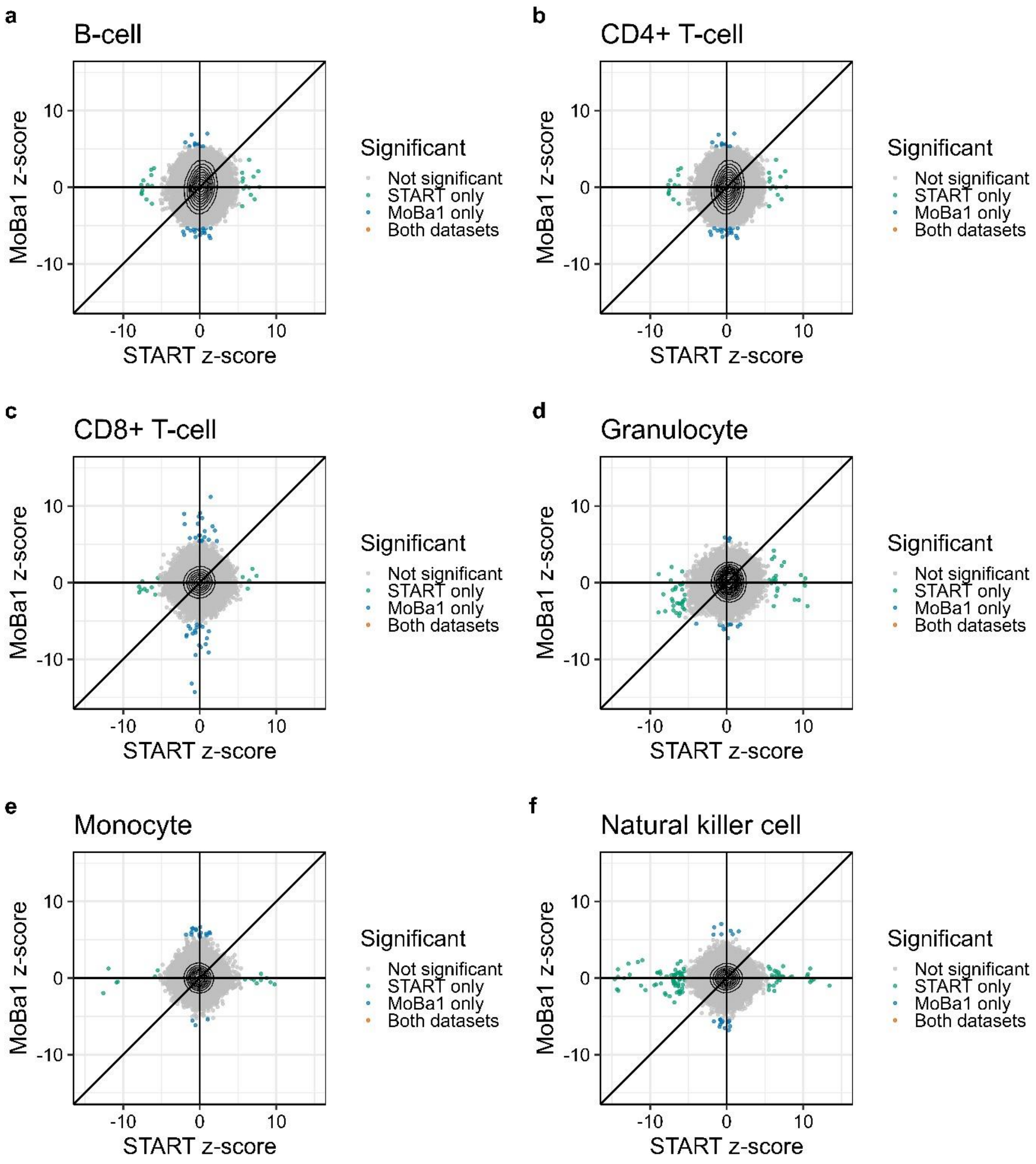


**Supplementary Figure 5. Comparison of cell-type specific CpGs associated with GA in the START (EPIC-based) and MoBa1 (450k-based) datasets.** Grey dots indicate nonsignificant associations, blue dots show CpGs significantly associated only in MoBa1 (pB < 0.05), and green dots CpGs significantly associated only in START (pB < 0.05). Black circles indicate the density of the points, increasing towards the axes crossing point. The x and y axes represent z-scores (i.e., coefficient estimate divided by the standard error) for START and MoBa1, respectively.

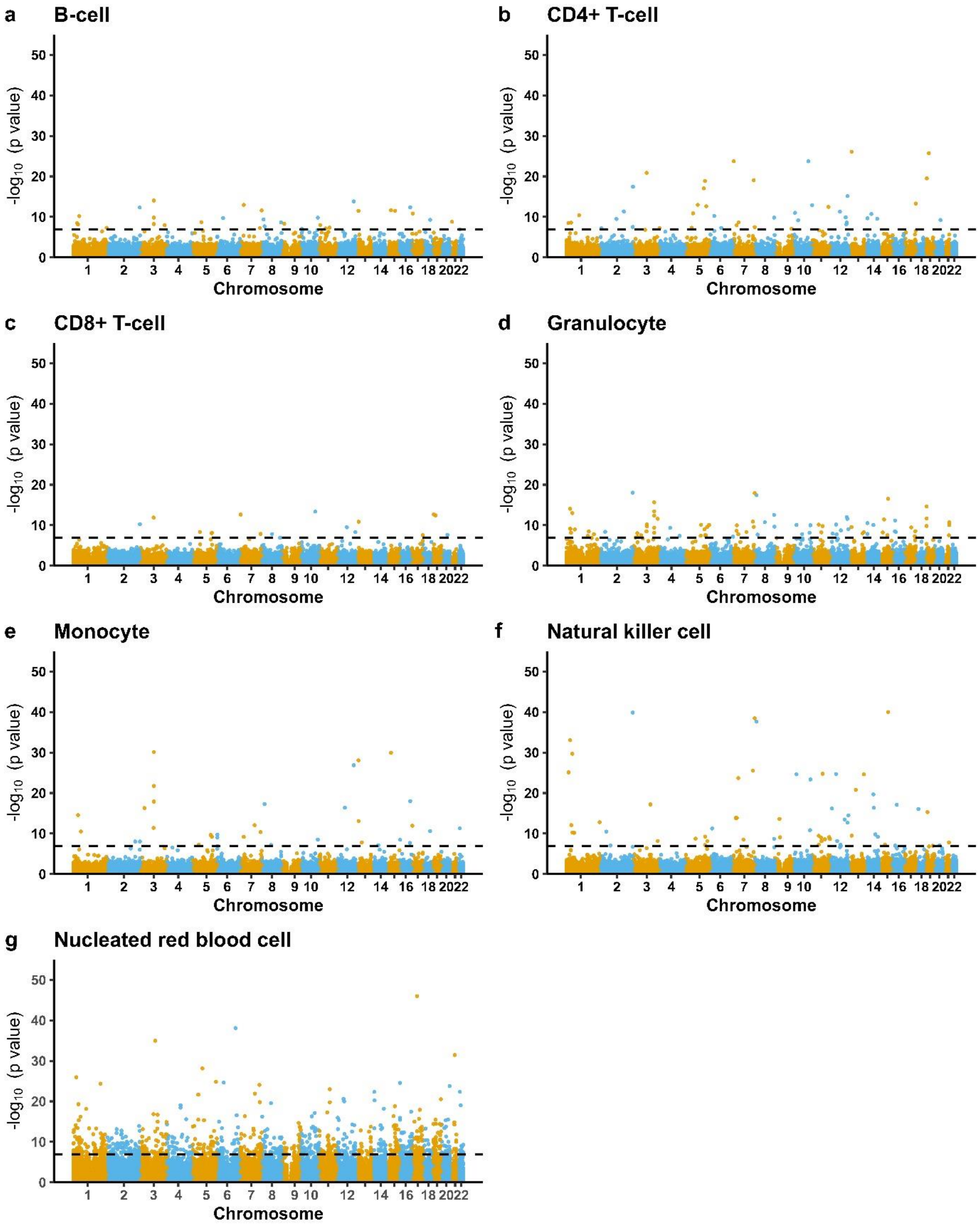


**Supplementary Figure 6. Manhattan plots of the epigenome-wide DNA methylation associated with GA in START identified using one-stage TCA.** Results are shown for each of the seven cell types. CpG loci are aligned on the x-axis according to their genomic coordinates. The y-axis represents the $-\log_{10}$ p values. The dashed black line denotes the Bonferroni-corrected genome-wide significance threshold ($p_B < 0.05$).

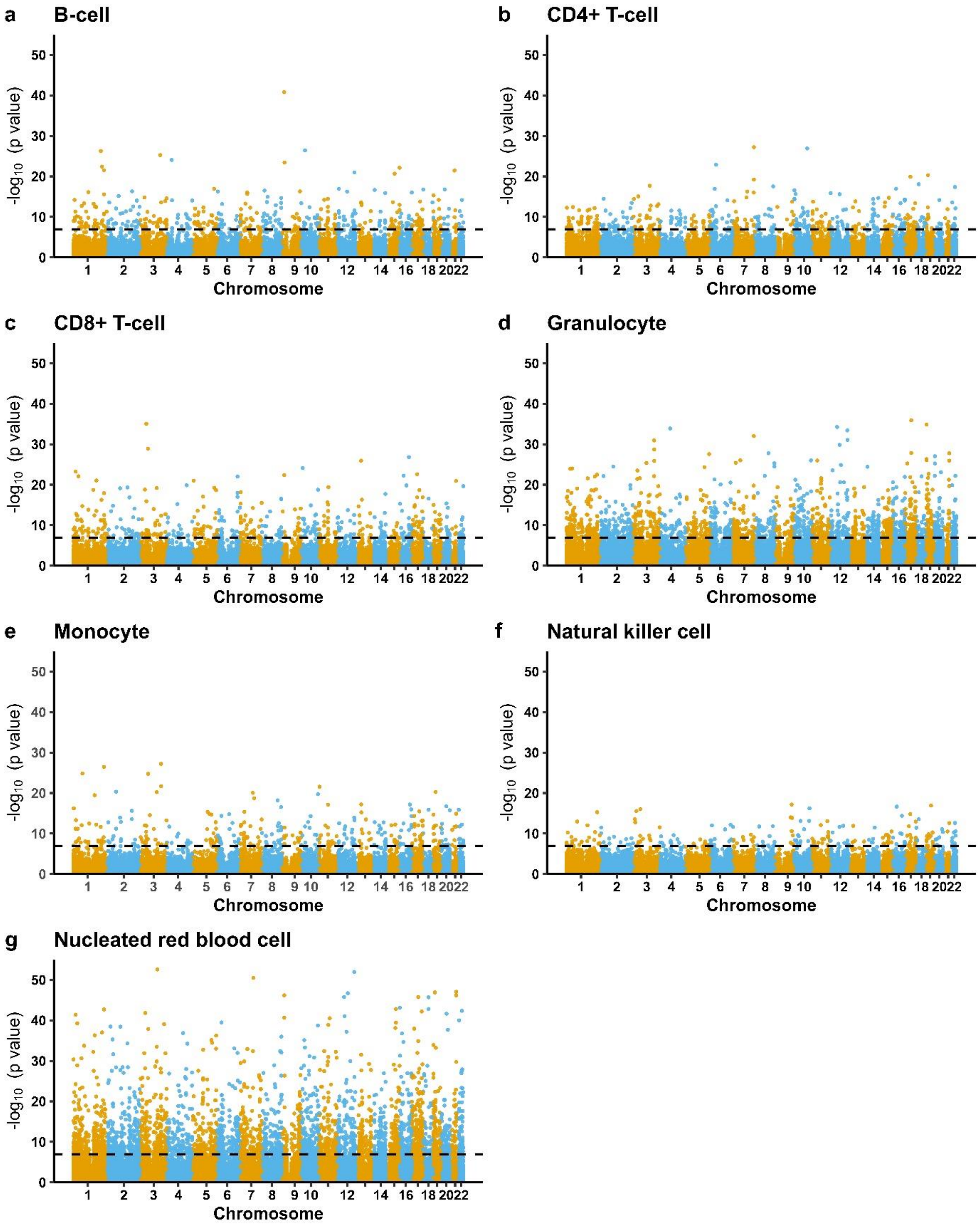


**Supplementary Figure 7. Manhattan plots of the epigenome-wide DNA methylation associated with GA in START identified using two-stage TCA.** Results are shown for each of the seven cell types. CpG loci are aligned on the x-axis according to their genomic coordinates. The y-axis represents the $-\log_{10}$ p values. The dashed black line denotes the Bonferroni-corrected genome-wide significance threshold ($p_B < 0.05$).

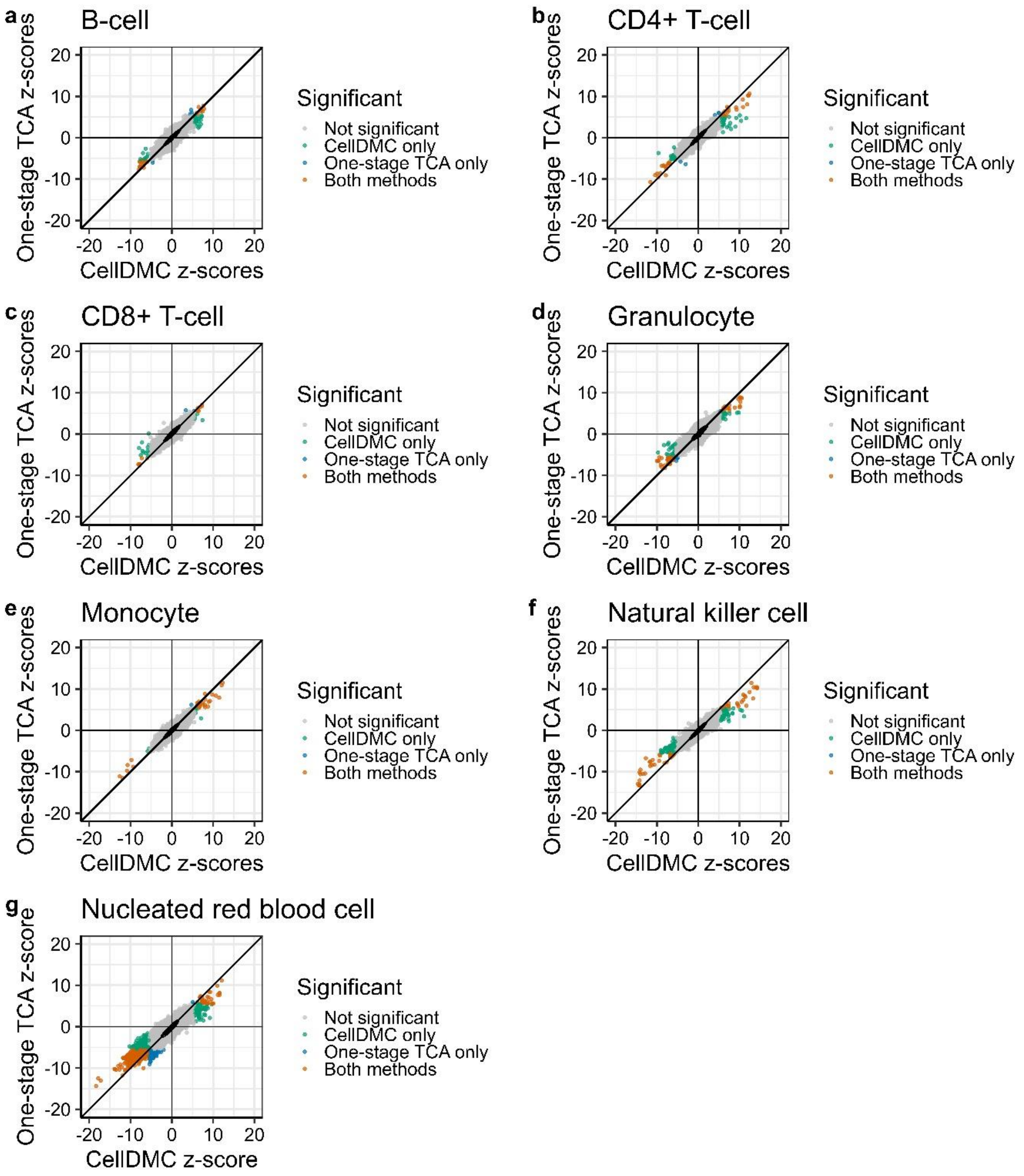


**Supplementary Figure 8. Comparison of cell-type specific CpGs associated with GA in the START dataset identified using CellDMC and one-stage TCA.** Grey dots indicate nonsignificant CpGs, blue dots CpGs that were only significant when one-stage TCA was used (pB < 0.05), green dots CpGs that were only significant when CellDMC was used (pB < 0.05), and orange dots CpGs that were significant by both methods (pB < 0.05). Black circles indicate the density of the points, increasing towards the axes crossing point. The x and y axes represent z-scores (i.e., coefficient estimate divided by the standard error) from CellDMC and TCA, respectively.

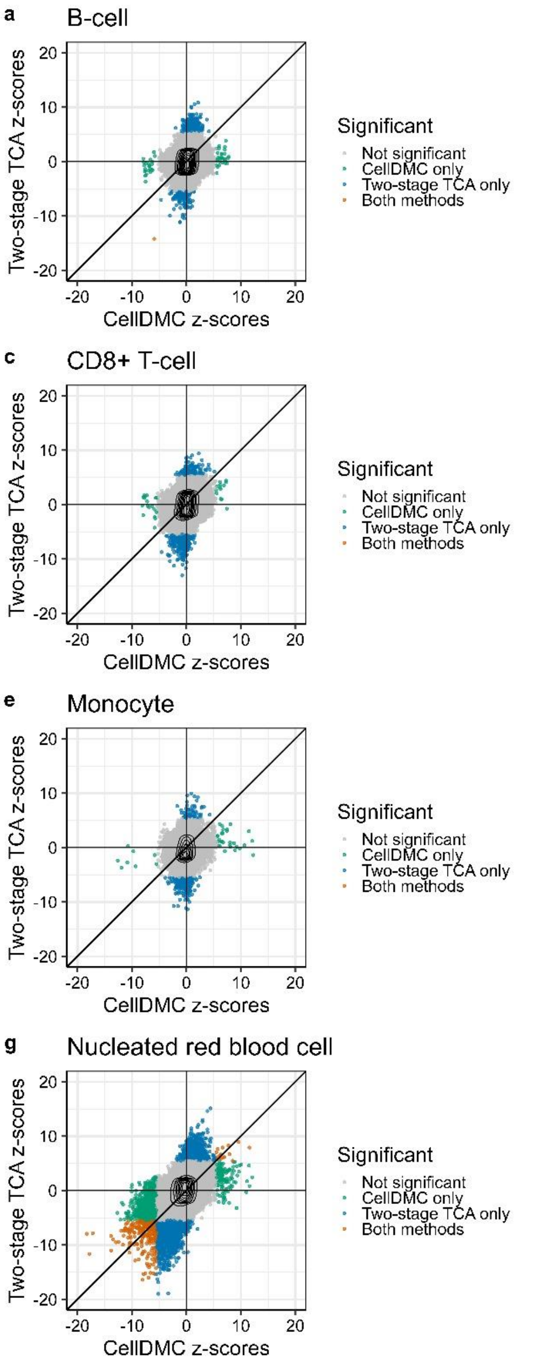


**Supplementary Figure 9. Comparison of cell-type specific CpGs associated with GA in the START dataset identified using CellDMC and two-stage TCA.** Grey dots indicate nonsignificant CpGs, blue dots CpGs that were only significant when two-stage TCA was used (pB < 0.05), green dots CpGs that were only significant when CellDMC was used (pB < 0.05), and orange dots CpGs that were significant by both methods (pB < 0.05). Black circles indicate the density of the points, increasing towards the axes crossing point. The x and y axes represent z-scores (i.e., coefficient estimate divided by the standard error) from CellDMC and TCA, respectively.

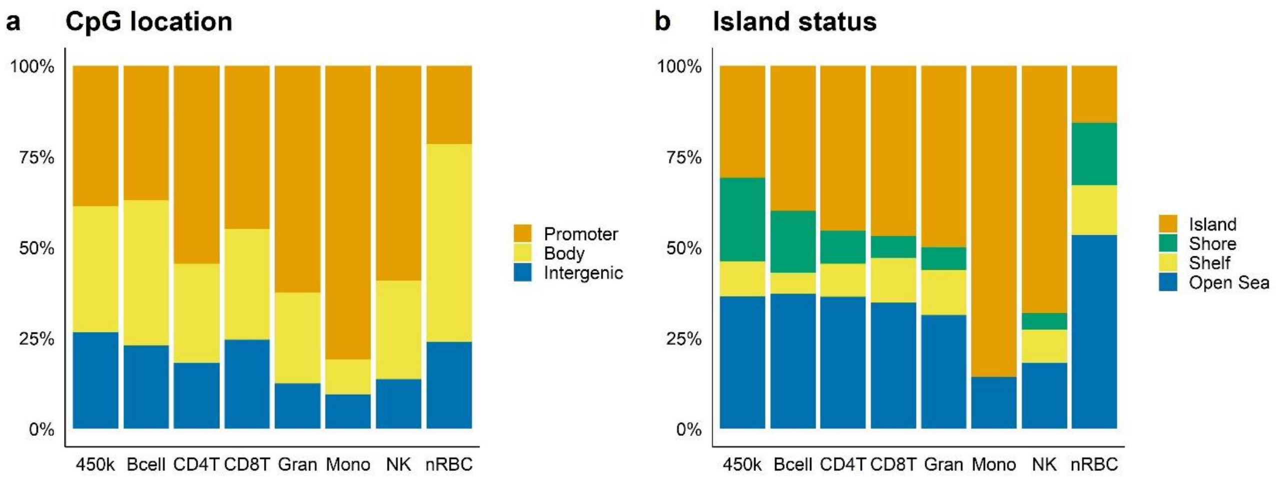


**Supplementary Figure 10. Position enrichment analyses of all the CpGs on the 450k array and those specifically associated with GA in each cell-type in the CellDMC analyses in MoBa1. a** The proportion of CpGs in promoter (orange), gene body (yellow), and intergenic (blue) regions. **b** The proportion of CpGs in CpG islands (orange), shores (green), shelves (yellow), and open sea (blue). Abbreviations: Bcell: B-cell, CD4T: CD4+ T-cell, CD8T: CD8+ T-cell, Gran: granulocyte, Mono: monocyte, NK: natural killer cell, nRBC: nucleated red blood cell.

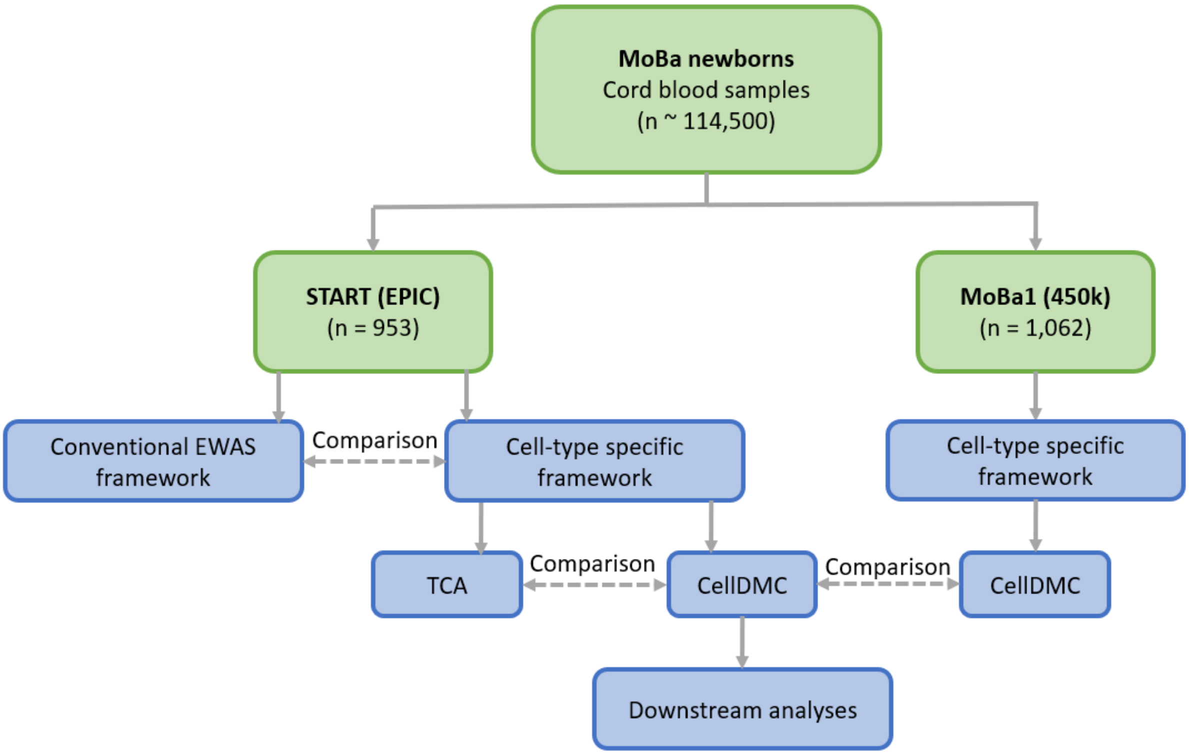


**Supplementary Figure 11. Analysis flow.** Two separate newborn cord-blood DNAm datasets from the MoBa cohort were used: START (generated using EPIC) and MoBa1 (generated using 450k). In START, we applied a conventional EWAS model as well as two different cell-type specific analyses using CellDMC and TCA, respectively. In MoBa1, we applied CellDMC only. We compared the results of the two different types of frameworks (conventional EWAS versus cell-type specific), the results of the two different cell-type specific methods (CellDMC and TCA), and the CellDMC-results for the two different datasets/array types (START/EPIC and MoBa1/450k). The datasets are marked in green and the methods in blue. Dashed arrows represent comparison between two sets of results.
