# Supplementary material for: Nucleated red blood cells explain most of the association between DNA methylation and gestational age
Source: Commun Biol. 2023 Feb 27;6:224. doi: 10.1038/s42003-023-04584-w (PMC9971030; doi:10.1038/s42003-023-04584-w)
Supplement: Supplementary file 2 — Description of Additional Supplementary Files [file 42003_2023_4584_MOESM2_ESM.pdf]

Description of Additional Supplementary Files

File name: Supplementary Data 1

Description: The source data behind Figure 1 in the paper.

File name: Supplementary Data 2

Description: Detailed information on results from the conventional EWAS model in START. The source data behind Figure 2a, Figure 3a and partly Figure 5 in the paper.

File name: Supplementary Data 3

Description: Detailed information on results from the CellDMC model in START. The source behind Figure 2b-h, Figure 3 b-h and partly Figure 5 in the paper.

File name: Supplementary Data 4

Description: Detailed information on results from the CellDMC model in MoBa1.

File name: Supplementary Data 5

Description: Detailed information on the comparison of CellDMC results between START and MoBa1. The source behind Figure 4 in the paper.

File name: Supplementary Data 6

Description: Detailed information on the results from the one-stage TCA model in START

File name: Supplementary Data 7

Description: Detailed information on the results from the two-stage TCA model in START

File name: Supplementary Data 8

Description: Detailed information on gene annotation of nRBC-specific CpGs associated with gestational age in START.

File name: Supplementary Data 9

Description: Detailed information on results from the GREAT analysis..

File name: Supplementary Data 10

Description: Examples of genes annotated to nRBC-specific CpGs associated with gestational age that are relevant for erythropoiesis.
