# Supplementary material for: Nucleated red blood cells explain most of the association between DNA methylation and gestational age
Source: Commun Biol. 2023 Feb 27;6:224. doi: 10.1038/s42003-023-04584-w (PMC9971030; doi:10.1038/s42003-023-04584-w)
Supplement: Supplementary file 13 — Reporting Summary [file 42003_2023_4584_MOESM13_ESM.pdf]

nature portfolio

Corresponding author(s): Kristine L. Haftorn

Last updated by author(s): Feb 2, 2023

Reporting Summary

Nature Portfolio wishes to improve the reproducibility of the work that we publish. This form provides structure for consistency and transparency in reporting. For further information on Nature Portfolio policies, see our Editorial Policies and the Editorial Policy Checklist.

Statistics

For all statistical analyses, confirm that the following items are present in the figure legend, table legend, main text, or Methods section.

n/a	Confirmed	
☐	☒	The exact sample size (*n*) for each experimental group/condition, given as a discrete number and unit of measurement
☐	☒	A statement on whether measurements were taken from distinct samples or whether the same sample was measured repeatedly
☐	☒	The statistical test(s) used AND whether they are one- or two-sided<br>*Only common tests should be described solely by name; describe more complex techniques in the Methods section.*
☐	☒	A description of all covariates tested
☐	☒	A description of any assumptions or corrections, such as tests of normality and adjustment for multiple comparisons
☐	☒	A full description of the statistical parameters including central tendency (e.g. means) or other basic estimates (e.g. regression coefficient) AND variation (e.g. standard deviation) or associated estimates of uncertainty (e.g. confidence intervals)
☐	☒	For null hypothesis testing, the test statistic (e.g. *F*, *t*, *r*) with confidence intervals, effect sizes, degrees of freedom and *P* value noted<br>*Give P values as exact values whenever suitable.*
☒	☐	For Bayesian analysis, information on the choice of priors and Markov chain Monte Carlo settings
☐	☒	For hierarchical and complex designs, identification of the appropriate level for tests and full reporting of outcomes
☐	☒	Estimates of effect sizes (e.g. Cohen's *d*, Pearson's *r*), indicating how they were calculated

Our web collection on statistics for biologists contains articles on many of the points above.

Software and code

Policy information about availability of computer code

Data collection	Raw data for the Illumina DNA methylation arrays were obtained using the proprietary software from Illumina (Genome Studio)
Data analysis	Methods and software used are stated in the methods section of the manuscript

For manuscripts utilizing custom algorithms or software that are central to the research but not yet described in published literature, software must be made available to editors and reviewers. We strongly encourage code deposition in a community repository (e.g. GitHub). See the Nature Portfolio guidelines for submitting code & software for further information.

Data

Policy information about availability of data

All manuscripts must include a data availability statement. This statement should provide the following information, where applicable:

- Accession codes, unique identifiers, or web links for publicly available datasets
- A description of any restrictions on data availability
- For clinical datasets or third party data, please ensure that the statement adheres to our policy

The data that support the findings of this study are available upon application to Norwegian Institute of Public Health (NIPH). Necessary ethical approvals will apply.

Human research participants

Policy information about studies involving human research participants and Sex and Gender in Research.

Reporting on sex and gender	In our study, information on newborn sex was collected from the national birth registry. Since newborn sex can sometimes be incorrectly reported, we also inferred it from the DNA methylation data. Based on this, one female was reclassified as male, and five males were reclassified as females. In total, 1024 males and 994 females were included in our analyses. Because sex can influence both DNA methylation and gestational age, we regarded it a possible confounder in our analyses, and therefore included it as a covariate.
Population characteristics	Gestational age ranged from 216-305 days (mean 280.1 days, SD±10.7 days) in the START dataset and 209-301 days (mean 279.8 days, SD ±10.8 days) in the MoBa1 dataset. The mean birthweight was 3657 g (SD±521 g) in START and 3643 g (SD±539 g) in MoBa1. Mean maternal age was 29.9 years (SD±4.7 years) in START and 29.9 years (SD±4.3) in MoBa1. In START, 478 (50%) of the mothers did not smoke before or during pregnancy; 245 (26%) smoked, but quit before pregnancy,;131 (14%) smoked, but quit early in pregnancy; and 102 (11%) continued smoking during pregnancy. In MoBa1, the corresponding counts were 522 (49%), 233 (22%), 154 (15%) and 153 (14%).
Recruitment	In our study, we have used two randomly selected subsamples of MoBa, which is a population-based pregnancy cohort study in which approximately 114,500 newborns, 95,200 mothers, and 75,200 fathers were recruited from all over Norway from 1999 to 2008. The mothers consented to participation in 41% of the pregnancies. Because recruitment was carried out during pregnancy, independently of gestational age at birth, and we assume that selection is not associated with DNA methylation, selection bias is not anticipated to impact our results.
Ethics oversight	This study was approved by the institutional review board at the Norwegian Institute of Public Health and by the Regional Ethics committee of South East Norway (#2017/1362). The establishment of MoBa and initial data collection was based on a license from the Norwegian Data Protection Agency and approval from the Regional Committees for Medical and Health Research Ethics. The MoBa cohort is now based on regulations related to the Norwegian Health Registry Act. All participants provided written informed consent.

Note that full information on the approval of the study protocol must also be provided in the manuscript.

Field-specific reporting

Please select the one below that is the best fit for your research. If you are not sure, read the appropriate sections before making your selection.

☒ Life sciences ☐ Behavioural & social sciences ☐ Ecological, evolutionary & environmental sciences

For a reference copy of the document with all sections, see nature.com/documents/nr-reporting-summary-flat.pdf

Life sciences study design

All studies must disclose on these points even when the disclosure is negative.

Sample size	This study included children who were singletons with a record in the Medical Birth Registry of Norway (MBRN) and whose mothers had returned the first MoBa questionnaire around pregnancy week 18. The sample selection is detailed in the analysis flow figure (Supplementary Fig. 9). We did not perform any sample size calculations but utilized the entire dataset that was available to us. Additionally, we are not aware of any a priori consensus regarding the minimum sample size required for an EWAS. Among the newborn who met the aforementioned inclusion criteria, we had 953 newborns in START and 1,062 newborns in MoBa1.
Data exclusions	We applied a quality control pipeline for data cleaning, where we removed cross-hybridizing probes and probes with low detection p-values. In addition, we excluded probes in which the last three bases overlapped with a SNP. We also visually inspected the RnBeads output of control probes and removed those with exceedingly low signals. Outliers with markedly different DNA methylation signals were also removed, and those probes that were removed from one batch due to poor quality were also removed in all subsequent batches.
Replication	To assess reproducibility of our findings, we applied the same method and statistical model as in the main model in the START dataset to a separate subsample of the MoBa cohort (the MoBa1 dataset) in which DNA methylation data was generated using a different array. We also applied a different cell-type specific method (TCA). The results were robust across the two different datasets, the two DNAm arrays, and the two different analysis methods.
Randomization	The measurement of DNA methylation on the Illumina microarrays was conducted in a randomized fashion through allocation of the newborns' DNA on random microarrays. Characteristics that potentially influence both the gestational age and the DNA methylation in newborns may confound the associations. We therefore controlled for these by including them as covariates in the model.
Blinding	We did not perform any blinding in this study as it was based on an ongoing cohort study (MoBa), with participants enrolled during pregnancy.

Reporting for specific materials, systems and methods

We require information from authors about some types of materials, experimental systems and methods used in many studies. Here, indicate whether each material, system or method listed is relevant to your study. If you are not sure if a list item applies to your research, read the appropriate section before selecting a response.

Materials & experimental systems

n/a	Involved in the study
☒	☐ Antibodies
☒	☐ Eukaryotic cell lines
☒	☐ Palaeontology and archaeology
☒	☐ Animals and other organisms
☒	☐ Clinical data
☒	☐ Dual use research of concern

Methods

n/a	Involved in the study
☒	☐ ChIP-seq
☒	☐ Flow cytometry
☒	☐ MRI-based neuroimaging
